# Profiles of GPs with high and low self-reported physician empathy—personal, professional, and antibiotic prescribing characteristics

**DOI:** 10.1186/s12875-022-01847-z

**Published:** 2022-09-20

**Authors:** Troels Kristensen, Charlotte Ejersted, Peder Ahnfeldt-Mollerup, Jens Søndergaard, Justin A. Charles

**Affiliations:** 1 DaCHE, Department of Public Health and Research Unit of General Practice, University of Southern Denmark, J.B. Winsløws vej 9, 5000 Odense C, Denmark; 2 Department of Public Health, Research Unit of General Practice, University of Southern Denmark, J.B. Winsløws Vej 9, 5000 Odense C, Denmark; 3 Department of Endocrinology, Odense University Hospital, J.B. Winsløws vej 4, 5000 Odense C, Denmark; 4 Department of Family Medicine and Public Health, University of California San Diego, 9500 Gilman Drive MC 0965, La Jolla, CA 92093 USA

**Keywords:** General practice, Physician empathy, Profiles of GPs, Extreme group analysis, Antibiotic prescribing, Bootstrapping

## Abstract

**Background:**

General Practitioners’ (GPs) professional empathy has been hypothesized to have substantial impact on their healthcare delivery and medication prescribing patterns. This study compares profiles of personal, professional, and antibiotic prescribing characteristics of GPs with high and low empathy.

**Methods:**

We apply an extreme group approach to a unique combined set of survey and drug register data. The survey included questions about demographic, professional, and antibiotic prescribing characteristics, as well as the Jefferson Scale of Empathy for Health Professionals (JSE-HP) to assess self-reported physician empathy. It was sent to a stratified sample of 1,196 GPs comprising 30% of the Danish GP population of whom 464 (38.8%) GPs responded. GPs in the top and bottom decile of empathy levels were identified. All intra- and inter-profile descriptive statistics and differences were bootstrapped to estimate the variability and related confidence intervals.

**Results:**

61% of GPs in the top decile of the empathy score were female. GPs in this decile reported the following person-centered factors as more important for their job satisfaction than the bottom decile: The Patient-physician relationship, interaction with colleagues, and intellectual stimulation. High-empathy scoring GPs prescribed significantly less penicillin than the low-empathy GPs. This was true for most penicillin subcategories. There were no significant differences in age, practice setting (urban vs. rural), practice type (partnership vs. single-handed), overall job satisfaction, or GP’s value of prestige and economic profit for their job satisfaction. The intra profile variation index and confidence intervals show less prescribing uncertainty among GPs with high empathy.

**Conclusions:**

This study reveals that high empathy GPs may have different personal, professional, and antibiotic prescribing characteristics than low empathy GPs and have less variable empathy levels as a group. Furthermore, person-centered high empathy GPs on average seem to prescribe less penicillins than low empathy GPs.

## Introduction

Physician empathy is a critical part of the physician–patient relationship and an important component of health care delivery in general practice and more broadly [1–3]. Empathy levels are heterogenous in the sense that each individual has a baseline degree of empathy that has the potential to increase with training or decrease based on environmental factors. Higher empathy levels among general practitioners (GPs) are associated with increased patient satisfaction, physician job satisfaction, physician self-esteem, decreased physician burnout, decreased risk of litigation, reduction in risk of medical errors and even improved patient health outcomes [4, 5]. However, both high and low empathy among GPs may have costs to society, which may be relevant to stakeholders [1, 6]. For instance, GPs with higher empathy may be constantly sensitive to others’ thoughts and feelings, which can interfere with their ability to act on their own thoughts and feelings [6]. In contrast, patients of lower empathy GPs may present less frequently to care, be less likely to adhere to treatment recommendations, be less satisfied and have worse health outcomes [7]. Based on this knowledge, it has become relevant to explore heterogeneity across empathy levels and related behavior [1]. One behavior worth investigating is antibiotic prescribing patterns of GPs [8]. Inappropriate prescription and related significant between-physician-variation in antibiotic prescribing patterns have been associated with high rates of antibiotic resistance and fluctuations in morbidity, mortality, and health care cost [9]. Some of the variation can be explained by factors like physicians’ attitudes [10]. Hence, an underlying reason for these factors can to some extent be physician empathy. Several have theorized that variation in empathy levels may play a role in antibiotic prescribing patterns [5, 11, 12]. Furthermore, empathy has been introduced as a basic concept that allows for understanding of behavior [13–15]. However, the features of GPs with the highest and lowest empathy and their antibiotic prescribing are not well profiled. To the best of our knowledge, no studies have investigated GPs with high and low empathy and only a few studies have explored empathy and antibiotic prescribing behavior [5, 11, 16, 17]. This study aims to make and compare profiles of personal, professional, and antibiotic prescribing behavior of GPs with the highest and lowest empathy scores. We hypothesize that high and low empathy GPs will differ in their antibiotic prescription frequency. More precisely, GPs with a higher degree of empathy would prescribe less antibiotics.

## Methods

This study is a combined questionnaire and register study and uses an extreme group design to analyze subgroups of a dataset of 464 GPs based on extreme level of a continuous empathy score with a range from 20–140 [18]. Our analysis is restricted to only using extreme observations for portraits in profiles of subgroups. The extreme groups were chosen as top and bottom deciles of the empathy score to balance opposing effects on statistical power of the desire to explore extreme groups versus the number of GPs in these groups. This strategy both allowed us to explore GPs with high and low empathy and achieve greater power in terms of lager differences between subgroup means (effect size).

The sample of data is from a 2017 GP survey sent by this research group as well as matched register data on the GPs’ antibiotic drug prescription from the Danish National Drug Register from 2017. The survey used the Jefferson Scale of Empathy for Health Professionals (JSE-HP) to measure physician empathy and included an addendum with questions about GPs’ demographic, professional, and job satisfaction characteristics.

### Jefferson Scale of Empathy for Health Professionals (JSE-HP)

The JSE-HP is a self-reported psychometric tool that measures cognitive and behavioral empathy by asking practitioners to rate their agreement with 20 statements on a 7-point Likert scale [19]. These 20 statements have been further divided into three components or subscales using factor analysis in previous studies [20, 21]. The three subscales are perspective taking (PT) (10 statements) which involves items related to “the physician’s view of patient’s perspective”, compassionate care (CP) (8 statements), which is defined as “a combination of empathy and sufficient degree of sympathy”, and walking in patient’s shoes (WPS) (2 statements) [20, 21]. The JSE-HP scores range from 20 to 140, with higher scores indicating a more empathic behavioral orientation [20]. The scale was created in English, but has been adapted to 55 languages, including Danish [4, 22]. Evidence of its convergent, discriminant, concurrent, and predictive validity, as well as internal consistency, test–retest reliability, and low social desirability bias is well-established among health professionals in the United States, and to varying degrees in international settings, including Denmark [19, 22].

### Survey addendum

The survey contained additional questions about GP’s demographic information, professional experience, and job satisfaction. GPs were asked how satisfied they are with their job and had five response options ranging from very unsatisfied to very satisfied. They were also asked to rank how much certain factors contributed to their job satisfaction on a 7-point Likert scale. These factors were physician–patient relationship, intellectual stimulation, interaction with colleagues, economic profit, and prestige.

### Survey sample

The web-based survey was distributed to a random, stratified sample of 1,196 Danish GPs practicing in Denmark in December 2016 and closed in January 2017. The sample was stratified by practice type and location. A more detailed description of this stratification can be found here [4].

#### Antibiotic data

The data on prescriptions of antibiotics from the included GPs were obtained through the Danish National Prescription Registry for the year 2017 after the GPs filled out the JSE-HP. The data from this registry included variables such as number of units of drug dispensed, and ATC code. The prescription registry data was merged with the empathy survey via the individual GP’s authorization number. The total number of yearly antibiotic prescriptions per GP was determined with this data.

#### Anatomical therapeutic chemical classification system codes

We extracted data using the category J01 (2 levels of specificity), antibiotics for systemic use according to the 2017 Danish Integrated Antimicrobial Resistance Monitoring- and Research Programme (DANMAP) and the 2013 categorization of broad and narrow spectrum antibiotics [23–25]. The Anatomical Therapeutic Chemical (ATC) codes included in this study are pencillins (J01C), other antibiotics (J01A, J01D, J01E, J01F, J01M and J01X) and antifungals (J02). The antibiotics were also categorized into “broad spectrum” and “narrow spectrum” antibiotics.

### Statistical analysis of profiles

The high-empathy group was defined as GPs who had empathy scores above the 90^th^ percentile, and the low-empathy group consists of all GPs who had scores below the 10^th^ percentile. The number of observations in the high and low empathy groups are by definition low in extreme group analysis and the underlying distributions of the empathy score often not known [26]. The rationale was to compare the most empathic versus the least empathic GPs while keeping a minimum of observations (N > 30) for testing inference. This approach was inspired by previous studies of GP characteristics and recommended use of extreme group analysis in pilot studies where the goal is to detect trends in samples [27]. Furthermore, Regional quality units in health care often have suggested GPs and other health care providers to use subgroup profiles to know strengths, areas of development and inspire reflection.

**Fig. 1:**
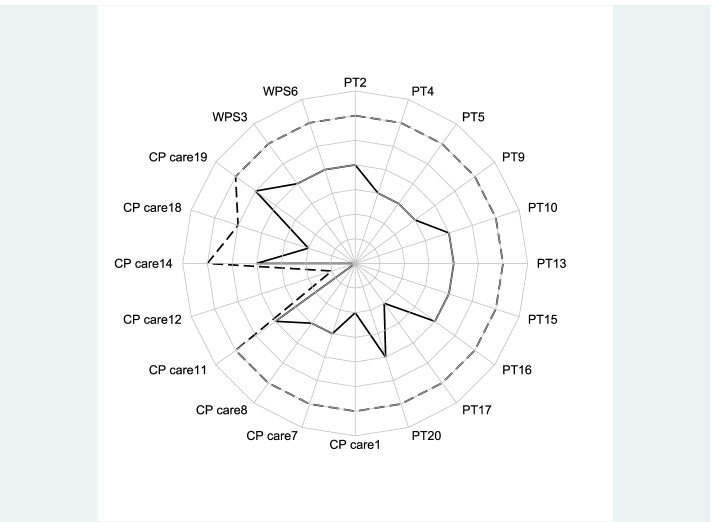
GP Scores in components of the JSE-HP for high and low empathy profiles. Scores from 1–7 for each of the 20 items on the JSE-HP scale. 1 at center and 7 at the periphery. Perspective taking (PTn), item *n* = 2,4,5,9,10,13,15–17,20. Compassionate care (CPn), item 1,7,8,11,12,14,18,19. Walking in patient’s shoes(WPSn:), item *n* = 3,6. Black dashed line: high empathy GPs Black solid line: Low empathy GPs. The abbreviated JSE items are: Understanding patients’ feelings influences treatment”(CP1), “Understanding makes patients feel better(PT2)”, “Viewing patients’ perspectives (WPS3)” “Understanding body language in communication” (PT4), “Sense of humor and clinical outcomes” (PT5), “Taking patients’ perspectives” (WPS6), “Attention to patients’ emotions” (CP7),”Attention to patients’ personal experiences” (CP8), “Standing in patients’ shoes” (PT9),”Understanding is therapeutic to patient” (PT10), Patient-physician emotional ties in medical treatment (CP11), “Life events in understanding physical complaints” (CP12),”Non-verbal cues and body language in understanding patients” (PT13),”Place of emotion in medical treatment” (CP14),” Empathy and clinical success” (PT15), “Understanding emotions in patient-clinician relationship” (PT16), “Thinking like patients for better care” (PT17), “Physician influenced by patients’ personal bonds” (CP18), “Enjoy literature and arts” (CP19) and “Empathy as a therapeutic factor” (PT20). The specific wording of the questions cannot be disclosed due copyright^4^

The profiles contained the following GP characteristics: Empathy scores, subcomponents of the empathy score, age, gender, practice type, and factors affecting job satisfaction. To compare the inter-group variation in prescribing of different drugs between the highest and lowest empathy groups a variation index was defined (90% percentile/10% percentile) and calculated [28]. The coefficient of variation was used to calculate intra-group variation across both high and low empathy groups and between GP characteristics [29]. A radar plot was created to visualize how high- and low-empathy GPs scored across all JSE-HP items and were further broken down by the PT, CP, and WPS subscales, as seen in Fig. 1. This type of plot depicts the average score (1–7) for the 20 items with the highest score placed on the outermost circle and the lowest score placed at the center.

We evaluated the difference between GP characteristics of the high- and low-empathy groups using the Mann–Whitney (Rank Sum Test) for independent samples both for continuous and ordinal variables, and the equality of proportions test for dichotomous variables such as gender (Table 1). The justification behind using the Rank Sum Test rather than the two-sample t-test is that the underlying subpopulations are not normally distributed. Next, we compared the average number of antibiotic prescriptions (derived from DANMAP data) made by the high- and low-empathy GPs using Mann–Whitney and T-test (Table 2).

**Table 1:** Profiles of high and low-empathy GPs: personal and professional characteristics

	**High-empathy GPs †**		**10th decile (*n* = 39)**		**Low-empathy GPs ‡**		**1st decile (*N* = 46)**		**Group difference measures**				
**Characteristics**	**Mean**	**[95% CI]**	**CV**	**[95% CI]**	**Mean**	**[95% CI]**	**CV**	**[95% CI]**	**VI**	**[95% CI]**	**Mean DIFF**	**[95% CI]**	***P*-value**
Total Empathy Score (20 items)	135.49	[134.41; 136.56]	0.015	[0.011;0.018]	97.28	[95.04; 99.53]	0.052	[0.037;0.0647]	1.39	[1.36;1.43]	38.21	[35.67;40.73]	< 0.0001***
PT subscore (10 items)^A^	67.77	[67.08;68.46]	0.027	[0.021;0.033]	47.3	[45.74;48.87]	0.092	[0.071;0.114]	1.43	[1.38;1.48]	20.47	[18.72;22.21]	< 0.0001***
CP subscore (8 items)^A^	53.92	[53.31; 54.54]	0.027	[0.018;0.036]	39.28	[37.99;40.58]	0.096	[0.074;0.117]	1.37	[1.33;1.42]	14.64	[14.64;16.05]	< 0.0001***
WPS subscore (2 items)^A^	13.79	[13.61; 13.98]	0.034	[0.017;0.051]	10.70	[10.04;11.36]	0.193	[0.151;0.235]	1.29	[1.21;1.367]	3.09	[2.41; 3.79]	< 0.0001***
**Demographics**													
Physician Age	56.33	[54.16; 58.51]	0.128	[0.104; 0.153]	55.91	[53.45;58.37]	0.138	[0.115;0.161]	1.01	[0.95;1.07]	0.42	[-2.91; 3.75]	0.7775
Male	38.50%	[23.09;53.83]			67.40%	[52.6;8.22]			0.57	[0.30;0.84]	-0.29	[-0.50; -0.08]	0.0076**
Female	61.50%	[46.17;76.91]			32.60%	[17.80;47.40]			1.89	[0.69;3.08]	0.29	[0.08;0.50]	0.0076**
**Practice Type**													
Urban	46.20%	[28.28;63.03]			54.30%	[37.00;71.71]			0.85	[0.43;1,27]	-0.08	[-0.33;0.16]	0.4515
Rural	17.90%	[6.37;29.53]			15.22%	[3.72;26.72]			1.18	[-0.74;3.10]	0.03	[-0.14;0.19]	0.7351
Mixed Urban/Rural	35.90%	[20.75;51.05]			30.40%	[15.06;45.81]			1.18	[-0.34;2.39]	0.05	[-0.16;0.27]	0.5934
Partnership	69.20%	[53.62;84.84]			63.04%	[47.21;78.88]			1.1	[0.66;1.54]	0.06	[-0.14;0.26]	0.5488
Non-Partnership	30.77%	[15.16;46.48]			37.00%	[21.12;52.79]			0.83	[0.24;1.43]	-0.06	[-0.29;0.16]	0.5488
**Experience**													
Years since GP specialization	20.82	[18.48;23.16]	0.365	[0.289; 0.440]	20.41	[17.81;23.01]	0.407	[0.326;0.487]	1.02	[0.85;1.19]	0.41	[-3.17;3.98]	0.8702
Years in present practice	17.62	[15.04;20.19]	0.488	[0.388; 0.587]	18.87	[13.47;24.27]	0.963	[0.414;1.511]	0.93	[0.64;1.23]	-1.25	[-7.52;5.02]	0.6556
**Job Satisfaction**													
Satisfied	87.20%	[77.05;97.30]			71.74%	[58.50;84.98]			1.22	[0.95;1.48]	0.15	[-0.01; 0.32]	0.0825
Neutral	10.26%	[1.30;19.22]			15.22%	[3.51;26.93]			0.68	[-0.45;1.80]	-0.05	[0.20; 0.10]	0.4971
Unsatisfied	2.56%	[-4.06; 9.18]			13.04%	[3.51;22.58]			0.2	[-1.08;1.47]	-0.10	[-0.23; 0.015]	0.0799
**Contribution of medical Practice factors to Job Satisfaction**^**C**^													
Physician–patient relationship	6.69	[6.51;6.87]	0.092	[0.049;0.134]	5.59	[5.33;5.84]	0.139	[0.1065;0.172]	1.2	[1.13;1.26]	1.11	[0.78; 1.42]	< 0.0001***
Intellectual stimulation	6.21	[5.93;6.48]	0.134	[0.105;0.164]	5.04	[4.76;5.33]	0.191	[0.131;0.252]	1.23	[1.15;1.31]	1.16	[0.76; 1.56]	< 0.0001***
Interaction with colleagues	6.03	[5.63;6.42]	0.200	[0.137;0.262]	4.70	[4.25;5.14]	0.307	[0.227;0.388]	1.28	[1.14;1.43]	1.33	[0.74; 1.92]	< 0.0001***
Economic profit	5.13	[4.74;5.52]	0.242	[0.173;0.310]	4.76	[4.42;5.10]	0.235	[0.183;0.287]	1.08	[0.97;1.19]	0.37	[-0.16; 0.89]	0.1377
Prestige	3.74	[3.27;4.22]	0.410	[0.333;0.486]	3.78	[3.42;4.14]	0.320	[0.228;0.412]	0.99	[0.83;1.15]	-0.04	[-0.65; 0.57]	0.5055

†: High-empathy GPs (10th decile). ‡ :  Low-empathy GPs (1st decile). PT: Perspective Taking, CP: Compassionate Care, WPS: Walking in the patient’s shoes. ^A^: The fraction of total score that each subscore contributes is the items shown in parenthesis out of the 20 items in the total empathy score, CV: Coefficient of variation = (SD/Mean); VI: Variation index = %ile>90 group† mean (*n *=39)/ %ile<10 group ‡ mean (*n *= 46). ^B^: The differences between the groups have been tested: For dichotomous variables, the equality of proportions was tested; For continuous or ordinal variables, the Mann-Whitney (ranksum) test was applied. For both types of test the *p*-values are reported *: *p* <0.05, **: *p* <0.01 ***: *p* <0.001. Furthermore, a 95% CI for the mean group differences have been estimated. In table 1, all reported confidence intervals are based on bootstrapping (1000 reps) based on the observations in the high and low empathy groups respectively. ^C^Values of scores on 7 point-scale

**Table 2:** Number of antibiotic prescriptions for high and low-empathy GPs

	**High-empathy GPs **† **(*N* = 39)**				**Low-empathy GPs **‡** (*N* = 46)**				**Group difference measures**					
**Antibiotic category:**	**Mean**	**[95% CI]**	**CV**	**[95% CI]**	**Mean**	**[95% CI]**	**CV**	**[95% CI]**	**VI**		**[95% CI]**	**Mean**	**[95% CI]**	**Ranksum**^**B**^
Penicillins:														
Extended spectrum JO1CA	108.77	[92.35;125.19]	0.48	[0.34;0.61]	136.87	[115.72;158.02]	0.50	[0.39;0.61]	0.79	[0.62;0.97]		-28.10	[-54.61; -1.59]	0.0249*
Beta-lactamase sensitive J01CE	111.92	[95.78;128.07]	0.45	[0.33;0.56]	158.24	[127.15;189.33]	0.63	[0.50;0.76]	0.71	[0.53;0.88]		-46.32	[ -81.42:-11.22]	0.0364*
Beta-lactamase resistant J01CF	32.95	[26.67; 39.22]	0.57	[0.36;0.78]	46.98	[38.75; 55.21]	0.56	[0.42;0.71]	0.70	[0.51;0.90]		-14.03	[-24.52; -3.54]	0.0019**
Combinations with beta lactamase inhibitors J01CR	20.56	[14.41; 26.72]	0.96	[0.74;1.17]	24.37	[19.02;29.72]	0.72	[0.58;0.87]	0.84	[0.51;1.17]		-3.81	[-12.16; 4.55]	0.1503
All penicillins: J01C	274.21	[237.76;310.65]	0.41	[0.30;0.52]	366.46	[310.21;422.71]	0.50	[0.39;0.61]	0.75	[0.59;0.91]		-92.25	[-159.53; -24.97]	0.0047**
**Non-penicillin antibiotics:**														
Tetrayclines J01A	15.77	[11.81; 19.73]	0.83	[0.60;1.06]	14.30	[10.26;18.35]	0.92	[0.62;1.22]	1.10	[0.67;1.53]		1.46	[-4.28; 7.21]	0.4933
Cephalosporins J01D	0.13	[ -0.01; 0.27]	3.66	[1.37;5.94]	0.13	[0.02;0.24]	3.07	[1.11;5.03]	0.98	[-1.71;3.68]		-0.00	[-0.18; 0.17]	0.6553
Sulfonamides J01E	42.64	[33.24; 52.04]	0.71	[0.57;0.86]	41.00	[31.67;50.33]	0.75	[0.59;0.91]	1.04	[0.68;1.40]		1.64	[-11.96; 15.24]	0.7843
Macrolides J01F	63.26	[46.24; 80.27]	0.87	[0.57;1.16]	71.28	[51.55;91.02]	0.90	[0.68;1.13]	0.89	[0.53;1.25]		-8.03	[-34.59;18.54]	0.524
Quinalones J01M	18.18	[12.08; 24.28]	1.10	[0.80;1.40]	22.0	[15.17;29.00]	0.97	[0.77;1.18]	0.82	[0.43;1.22]		-3.91	[-13.28; 5.46]	0.3021
Other antibiotics J01X	13.87	[9.31;18.44]	1.07	[0.84;1.31]	13.74	[8.66;18.81]	1.15	[0.74;1.56]	1.01	[0.51;1.51]		0.13	[-6.71; 6.98]	0.846
All other antibiotics	153.85	[120.77;186.93]	0.70	[0.50;0.90]	162.54	[126.53;198.55]	0.71	[0.57;0.86]	0.95	[0.64;1.25]		-8.69	[-58.83;1.43]	0.88
**Antifungals, J02**	36.95	[29.48;44.42]	0.66	[0.52;0.80]	38.04	[29.94;46.15]	0.68	[0.49;0.87]	0.97	[0.68;1.26]		-1.09	[-11.90; 9.71]	0.7871
**Total all antibiotics:**	427.97	[366.07;489.88]	0.46	[0.37;0.56]	528.78	[443.46;614.11]	0.52	[0.42;0.63]	0.81	[0.63;0.99]		-100.81	[-207.84; 6.201]	0.0643
Narrow spectrum (N)	264.59	[223.92, 305.26]	0.49	[0.38;0.60]	331.07	[273.35;388.78]	0.57	[0.44;0.69]	0.80	[0.61;0.99]		-66.48	[-138.16; 5.21]	0.1078
Broad spectrum (B)	163.38	[138.94, 187.82]	0.48	[0.37;0.56]	197.72	[167.08;228.36]	0.50	[0.41;0.64]	0.83	[0.64;1.01]		-34.33	[-73.89; 5.23]	0.0751

† : High-empathy GPs (10th decile). ‡ : Low-empathy GPs (1st decile). CV: Coefficient of variation = (SD/Mean); Variation index (VI) = %ile>90 group† mean (*n *= 39)/ %ile<10 group ‡ mean (*n *= 46). ^B^: The differences between the groups have been tested: The Mann-Whitney (ranksum) test was applied. ^*^: *p* <0.05, ^**^: *p* <0.01 ^***^: *p* <0.001. Furthermore, 95% CIs for the mean group differences have been estimated. All reported confidence intervals are based on bootstrapping (1000 reps) of the observations in the high and low empathy groups respectively. N: Narrow spectrum antibiotic was defined via the following ATC-codes: J01CE, J01CF, J01DB, J01DF, JO1EA, J01EB, J01FA, J01FF, J01XA, J01XC, J01XD, J01XE, J01XX. B: Broad spectrum antibiotics included: J01AA, J01CA, J01CR, J01DC, J01DD, J01DH, J01EE, J01GB, J01MA, J01MXB

### Bootstrapping techniques

To estimate uncertainty around the applied statistics such as the group mean, coefficient of variation and variation index, this study uses bootstrapping techniques to estimate 95% confidence intervals (CIs) [30, 31]. Bootstrapping allows these statistics to be calculated based on the entire sample rather than a parametric approach based on an unknown but assumed non-normal distribution. This was done to get a sense of what the true unknown distribution is in the two groups. Finally, the bootstrapped difference of mean across high and low empathy was used to perform parametric tests of the difference in mean characteristics. The latter allowed us to compare with the parametric tests mentioned above.

## Results

The response rate was 39% (*n* = 464) of 1196 survey recipients. Of the respondents 39 GPs scored at the high-end 10^th^ decile while 46 GPs scored at the low-end of the JSE-HP scale. The radar plot in Fig. 1 shows the average score on the Likert scale (1–7) for the 20 items of the JSE-HP, broken down by subscale (PT, CP, and WPS) for groups of GPs with the highest empathy (dotted-line) and lowest empathy (black solid line) scores. The abbreviated JSE-HP items are reported under Fig. 1.

The high empathy GPs averaged 7 on all scale items except for CP12 and CP18. Low empathy GPs (solid line) scored lowest on items 1, 12, 17 and 18, and highest on CP19 and PT20. Thus, high empathy GPs scored relatively higher than low empathy GPs in PT subcomponents compared to CP. The scores of GPs in highest decile scores were largely consistent, while those in the lowest decile group were more varied across all subgroups as indicated by the coefficient of variations and confidence intervals in Table 1 below. The items with the greatest difference between high and low empathy groups were CP1 and PT17. Both high empathy and low empathy GPs scored disproportionately low in CP12 “Life events in understanding physical complaints” which indicates that asking patients about what is happening in their personal lives is helpful in understanding their physical complaints.

Table 1 displays profiles of the group of GPs with the highest decile (90^th^ percentile) and lowest decile (10th percentile). This includes descriptive statistics on the empathy score and its’ subcomponents, as well as personal and professional characteristics for the two groups. The mean empathy score of high empathy GPs (135) was 36 score points greater than the score of 99 for low empathy GPs (*p* < 0.001). The scores were significantly higher in the high empathy group across all three subscales.

Overall, the intra-group variation measured by the coefficients of variation and related 95% confidence intervals was over three times higher among low empathy GPs, compared to high- empathy GPs for total score and among all subcomponents.

Among the personal characteristics, the majority of the high empathy group was female (61.5%), whereas the majority of the low empathy group was male (67.4%) (*p* = 0.0076). There was no difference in age across high and low profiles. Of the professional characteristics, there were no differences between the two groups with respect to practice type, years since completion of GP training, and years in present practice. While the difference in overall job satisfaction between the groups was not statistically significant, there was a trend towards higher job satisfaction in the top decile with high empathy GP’s (*p* = 0.0825).

The GPs in the top decile placed significantly greater value on the contribution of the physician–patient relationship (6.69 vs. 5.59), intellectual stimulation (6.21 vs. 5.04) and interaction with colleagues (6.03 vs. 4.70) to their job satisfaction (all *p* < 0.0001). There was no intergroup difference with respect to the contribution of prestige and profit.

The variation index, which reflects intergroup variation, was relatively high for gender, total empathy score and its subcomponents, overall job satisfaction, contribution to job satisfaction from the physician–patient relationship, intellectual stimulation and interaction with colleagues. In contrast, there was relatively low intergroup variation with respect to the GP’s age, practice type, experience, contribution to job satisfaction from economic profit and prestige. Overall, the variation index and related 95% confidence intervals were lower in GPs with the highest empathy than those with the lowest empathy across personal and professional GP characteristics. The antibiotic prescribing profiles are shown in Table 2.

### Antibiotics prescribing profiles

Overall, the high-empathy GPs made 19% fewer antibiotic prescriptions per year than the low empathy group (428 vs. 529 prescriptions).

### Penicillins profiles

The most frequently prescribed antibiotic was the group of penicillins (JO1C) which represents 64% (high empathy profile) and 69% (low empathy profile) of all types of antibiotics as shown in Table 2. High-empathy GPs made 92 fewer prescriptions among all types than the low-empathy GPs. Low empathy GPs also prescribe relatively more antibiotics in most penicillin subcategories, except for those Combinations with beta lactamase inhibitors (B) J01CR. For these categories the measured intergroup variation index range in terms of the mean penicillin prescribing was between 0.70 and 0.84. The variation index was highest for penicillins with extended spectrum (JO1CA) 0.79, (J01CR) 0.84 and lowest for beta-lactamase sensitivity and beta-lactamase resistant penicillins (J01CE & J01CF) 0.70–0.71 belonging to the group of narrow spectrum antibiotics. In addition, the test of differences shows a significant mean difference between groups for the narrow spectrum penicillins (J01CE & J01CF) and one of the two broad spectrum penicillins.

### Non-penicillin profiles

In most cases, this group of antibiotics are used after bacterial culture (and known resistance pattern) and thus based on a more precise and stringent diagnosis.

Table 2 shows that there were no differences in prescribing of non-penicillin antibiotics across the high versus low empathy groups.

### Narrow versus broad spectrum

Calculated size effects indicates that low empathy GPs both prescribed narrow and broad spectrum antibiotics more often but this trend was not significant (*p* < 0.05).

## Discussion

This study contributes to the literature in several ways. First, we explore personal and professional profiles of GPs with high and low empathy scores. Second, we break down the empathy scores for the high and low empathy groups into the three factors of the JSE-HP: 1) perspective taking, 2) compassionate care and 3) walking in patient’s shoes. This breakdown permits us to visualize and explore how these components contribute to the variation and difference between the high and low empathy profiles. Third, we link the empathy scores of each individual GP to their antibiotic prescribing patterns. To the best of our knowledge this is the first study that creates profiles of both personal, professional and antibiotic prescribing for GPs with high and low empathy.

### Personal and professional characteristics

The top-decile of high empathy scoring GPs was composed of more females than men. This contrasts with findings from a previous study using the same sample that indicated no link between gender and the empathy score, when the score was treated as a dichotomous variable [4]. It is therefore possible, that most of the gender variation in empathy happens at the extreme levels of empathy.

On the one hand, the female bias towards higher empathy in this study is not surprising. Gender differences in empathy have been observed in other studies, with women having higher levels of empathy [32]. Female medical students and physicians alike also have been documented to have higher empathy levels, especially when measured with the JSE-HP used in this study [33–35]. This may be because women have been shown to exhibit a higher level of empathetic concern than men and have a generally more “empathizing” behavioral style than men do [36–38]. On the other hand, men and women score similarly in most individual components that make up empathy, such as perspective-taking, ability to identify and describe feelings, and altruistic behavior [37, 39]. This has been theorized to be a result of women tending to more readily report empathetic experiences or to meet societal expectations to be more empathic [40]. Finally, there was no difference in the average age between the high and low empathy groups, which is consistent with a prior study showing no relationship between age and GP empathy [4].

High-empathy GPs placed greater value on interaction with colleagues, the physician–patient relationship, and intellectual stimulation than did low-empathy GPs. These factors are well established as important contributors to physician job satisfaction [41]. This suggest that high empathy GPs are more person-centered and place greater value on intellectual stimulation than low empathy GPs. Given the strong relationship between empathy, communication and patient-centered care, it is unsurprising that more empathic GPs would more greatly value their interactions with patients and colleagues [42]. Additionally, people with higher intelligence levels have been shown to have more emotional intelligence and empathy [43].

The lowest empathy GPs were more heterogeneous among empathy scores, demographic, professional, and antibiotic prescribing patterns. One reason may be that GPs in Denmark operate in heterogenous private businesses, organizations that tend to invoke the use of economic schema, which prioritize rationality, efficiency, and self-interest [44]. This cognitive framework, when activated by GPs, can result in dampening of empathy. The degree to which GPs utilize this schema likely varies, which may contribute to variation in GP empathy in this group [44]. Excess empathy can result in compassion fatigue and have harmful effects for GPs, such as increased stress or depression [45].

### Subcomponent of the empathy scores for the high versus low empathy GPs

The highest empathy GPs outscored the lowest empathy GPs across all subcomponents of the JSE-HP (CC, PT, and WPS). Scores on the PT and CC subscales contributed more to the overall score than did WPS. This is because the PT and CC subscales contain most of the items of the 20-item scale. Therefore, the largest component of the difference between high and low empathy GPs was from variation in scores on the perspective taking subcomponent.

### High and low empathy profiles and antibiotic prescribing

In general, the JSE-HP score among Danish GPs in the extreme groups were high. The scores of high-empathy GPs were close to the maximum JSE-HP score (140), whereas the low-empathy JSE-HP scores (97) were in the middle of the scale, rather far from the minimum score (20). Overall, the characteristics of the GPs in the low empathy profile was more heterogenous and varied than for GPs in the high empathy group.

The behavior of prescribing penicillin and narrow-spectrum penicillinase-resistant penicillin is different from prescribing broad-spectrum antibiotics. The narrow-spectrum antibiotics are prescribed to a greater extent according to culture and resistance pattern. As described in Table 2, this study find that high empathy GPs prescribe less (34%) penicillin than the low empathy group. Practitioners with a degree of empathy may prescribe less penicillin as they take better time to explain, meet the patient’s fears and expectations, and evaluate antibiotic choice in their community with reference to local resistance patterns [12]. A likely explanation may be that high empathy GPs better identify patient’s concerns and expectations and are able to contextualize the patient’s infection in the community. For instance, a low-empathy GP may prescribe unnecessary antibiotics because it is easier for the GP to follow a patient’s request and expectations rather than spend time exploring why a patient feels they need antibiotics and compassionately explaining the rationale for not prescribing antibiotics.

However, it should be noticed that a range of other reasons for GPs to prescribe antibiotics could explain the observed difference across profiles. GPs may be more likely to prescribe broader spectrum antibiotics for patients who are older or have more comorbidities. Additional reasons could be limited consultation time or ability to discuss utility, risks and benefits of antibiotics, preserving GP–patient relationships, medicolegal reasons, or risk perception about the severity of the illness among others [46]. Overall, this potential link may be for the benefit of patients served by high empathy GPs. For instance, in terms of lower drug costs and fewer resistant bacteria.

Inappropriate prescription of broad-spectrum antibiotics without culture and sensitivities can increase antibiotic resistance, which has known harms [47]. Therefore, it is useful to know that there were no prescribing differences between the groups for broad-spectrum penicillins and non-penicillin antibiotics, which may reflect appropriate use of clinical guidelines.

### Strength & limitations

#### Advantages

The applied extreme group analysis has the advantage that it can be used to explore profiles of GPs with high and low physician empathy and related characteristics that may be useful to learn about extreme group´s antibiotic prescribing behavior, generate hypothesis and inspire reflection. In particular, the applied methodology has helped us explore elements of antibiotic prescribing which may serve as the basis for further scientific studies and inspire policy making related to antibiotic prescribing and interventions targeted to promote more careful prescribing.

The applied split on low decile (10^th^ percentile) versus top decile (90^th^ Percentile) empathy scores rather than the choice of larger groups (e.g., quartiles) has the benefit that it allows us to create relatively extreme profiles of empathy corresponding to larger group difference between means (the effect size) that causes statistical power to increase. Furthermore, the applied subgroups keep a minimum of observations in each group (*n* > 30) for inference.

The sample of 464 GPs was expected to be representative of the total population of Danish GPs (3436 GPs) and included all antibiotic prescriptions made by these GPs in 2017.The applied extreme group design is well-suited for profiles of extreme groups and exploratory hypothesis generation in pilot studies where it can enhance the detectability of size effects and interaction effects [18]. This means, the approach focuses on extreme observations to cater to asymmetry. In addition, the extreme design requires no subjective methodological assumptions and only includes two subgroups to reduce the multiple comparisons problem.

#### Disadvantages

Extreme group analysis is faced with a trade-off between the proportion of the scores distribution which should be included into the extreme groups in terms of statistical power versus group mean differences (effect size). The nature of these opposing effects on power makes it impossible to both select extreme groups and achieve high statistical power at the same time. Therefore, portraits of extreme groups, by definition, mainly focuses on the extreme group element rather than the number of subgroup observations, knowing that the cost is reduced statistical power for inference [48].

Another disadvantage is that it was not possible to sample directly from extreme empathy groups a priori. This means that the statistical power of tests between the high and low empathy groups will often be reduced compared to specific sampling from extreme groups. Another disadvantage is that the extreme group design, in this study, is based on a limited subsample size. This increases the risk of both false positive and false negative findings, which may result in insufficient statistical power to confirm hypothesis [18]. However, there is a finite number of GPs and therefore, the present extreme group design cannot be based on far larger samples.

The Danish National Prescription Registry allowed us to capture a complete data sample for the patients and GPs in our study, as all prescriptions made in Denmark must go through this registry. However, because GPs sometimes cover for other colleagues, we do not know if all patients who received prescriptions from a certain GP are on that GP’s patient panel or not. This is a limitation because GPs may make different decisions for patients who they are less familiar with. Still, GPs predominantly write prescriptions for their own patients. In future research, we hope to be able to use GP list size data to calculate prescribing rates per patient and explore potential differences in prescribing behavior among GPs for their own patient panel compared to those from other GPs’ panels, such as those covered outside of regular office hours.

Knowledge of and attention to high and low empathy GPs may help health care system stakeholders to cultivate desired levels of empathy among GPs and thus influence their professional behavior to provide the best and most accurate service to patients.

## Conclusion

This study reveals that high empathy GPs may have different personal, professional, and antibiotic prescribing characteristics than low empathy GPs and have less variable empathy levels as a group. Furthermore, person-centered high empathy GPs on average seem to prescribe less penicillins than low empathy GPs.

## Data Availability

All data are available from the Danish national registers. https://sundhedsdatastyrelsen.dk/da/english/health_data_and_registers Due to ethical and legal issues, the raw data cannot be made publicly available. However, all interested readers may request additional information from corresponding author Troels Kristensen.
